# 4-Butyl-1-(2,3,4-tri-*O*-acetyl-β-l-fuco­pyranos­yl)-1*H*-1,2,3-triazole

**DOI:** 10.1107/S1600536809028700

**Published:** 2009-07-25

**Authors:** Abdul-Basit Alhassan, Peter Norris, Matthias Zeller

**Affiliations:** a Department of Chemistry, Youngstown State University, 1 University Plaza, Youngstown, OH 44555-3663, USA

## Abstract

The title compound, C_18_H_27_N_3_O_7_, was synthesized by Cu^I^-catalysed coupling of an azide with an alkyne as part of a study into the synthesis of *N*-glycosyl-1,2,3-triazoles. The crystal structure confirms the selective formation of the β-conformer of the pyran­ose *N*-glycoside, thus confirming the retention of stereochemistry during heterocycle formation with the *N*-glycosyl triazole group occupying the equatorial position at the anomeric C atom. The structure exhibits two crystallographically independent mol­ecules (*A* and *B*) with essentially identical conformations with a weighted r.m.s. deviation of only 0.09 Å. The mol­ecules are arranged in layers with hydro­phobic and more polar sections built from the butyl triazole units on the one hand and the more polar moieties dominated by the carbohydrate units on the other. Within the polar layers, inter­molecular inter­actions are dominated by a three-dimensional network of weak C—H⋯O hydrogen bonds with the acetyl keto O atoms as the hydrogen-bond acceptors. The triazole units inter­act with each other *via* C—H⋯N hydrogen bonds which connect the mol­ecules into two infinite chains of mol­ecules made up of either *A* mol­ecules or *B* mol­ecules that stretch parallel to each other along [100]. Between the butyl groups no directional inter­actions are observed.

## Related literature

For background information on *N*-glycosidic mimics of naturally occurring carbohydrates, see: Norris (2008 ▶); Temelkoff *et al.* (2006 ▶). For details of the synthesis of the carbohydrate starting material used, see: Zhang *et al.* (2007 ▶).
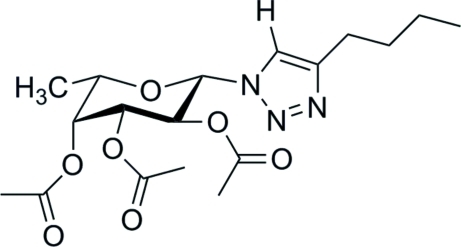


## Experimental

### 

#### Crystal data

C_18_H_27_N_3_O_7_
*M* *_r_* = 397.43
Triclinic, 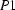

*a* = 5.5173 (3) Å
*b* = 7.7442 (4) Å
*c* = 24.1013 (13) Å
α = 94.507 (1)°
β = 96.151 (1)°
γ = 91.227 (1)°
*V* = 1020.22 (9) Å^3^
*Z* = 2
Mo *K*α radiation
μ = 0.10 mm^−1^
*T* = 100 K
0.36 × 0.35 × 0.09 mm

#### Data collection

Bruker SMART APEX CCD diffractometer
Absorption correction: multi-scan (*SADABS* in *SAINT-Plus*; Bruker, 2003 ▶) *T*_min_ = 0.867, *T*_max_ = 0.991
10512 measured reflections
5041 independent reflections
4839 reflections with *I* > 2σ(*I*)
*R*_int_ = 0.020

#### Refinement

*R*[*F* ^2^ > 2σ(*F* ^2^)] = 0.046
*wR*(*F* ^2^) = 0.120
*S* = 1.11
5041 reflections
515 parameters
3 restraints
H-atom parameters constrained
Δρ_max_ = 0.42 e Å^−3^
Δρ_min_ = −0.21 e Å^−3^

### 

Data collection: *SMART* for WNT/2000 (Bruker, 2002 ▶); cell refinement: *SAINT-Plus* (Bruker, 2003 ▶); data reduction: *SAINT-Plus*; program(s) used to solve structure: *SHELXTL* (Sheldrick, 2008 ▶); program(s) used to refine structure: *SHELXTL*; molecular graphics: *SHELXTL* and *Mercury* (Macrae *et al.*, 2008 ▶); software used to prepare material for publication: *SHELXTL* and *publCIF* (Westrip, 2009 ▶).

## Supplementary Material

Crystal structure: contains datablocks global, I. DOI: 10.1107/S1600536809028700/fl2257sup1.cif

Structure factors: contains datablocks I. DOI: 10.1107/S1600536809028700/fl2257Isup2.hkl

Additional supplementary materials:  crystallographic information; 3D view; checkCIF report

## Figures and Tables

**Table 1:** Hydrogen-bond geometry (Å, °)

*D*—H⋯*A*	*D*—H	H⋯*A*	*D*⋯*A*	*D*—H⋯*A*
C1*B*—H1*B*⋯O3*B*^i^	1.00	2.53	3.362 (3)	141
C2*A*—H2*A*⋯O3*A*	1.00	2.26	2.701 (3)	105
C2*B*—H2*B*⋯O3*B*	1.00	2.26	2.698 (3)	105
C3*A*—H3*A*⋯O3*A*^ii^	1.00	2.32	3.214 (3)	149
C3*B*—H3*B*⋯O3*B*^i^	1.00	2.27	3.165 (3)	148
C4*A*—H4*A*⋯O5*A*	1.00	2.56	3.046 (3)	110
C4*A*—H4*A*⋯O7*A*	1.00	2.23	2.682 (3)	106
C4*B*—H4*B*⋯O5*B*	1.00	2.57	3.067 (3)	110
C4*B*—H4*B*⋯O7*B*	1.00	2.21	2.670 (3)	106
C7*A*—H7*A*⋯N3*A*^ii^	0.95	2.39	3.308 (4)	161
C7*B*—H7*B*⋯N3*B*^i^	0.95	2.40	3.313 (4)	161
C14*A*—H14*A*⋯O1*A*^iii^	0.98	2.47	3.377 (3)	154
C14*B*—H14*D*⋯O1*B*^iii^	0.98	2.35	3.261 (3)	155
C16*A*—H16*A*⋯O7*B*^iii^	0.98	2.41	3.295 (4)	150
C16*B*—H16*F*⋯O7*A*	0.98	2.51	3.401 (4)	150

Symmetry codes: (i) 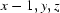; (ii) 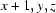; (iii) 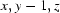.

**Fig. 1.**
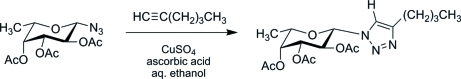
Synthesis of the title compound.

**Fig. 2.**
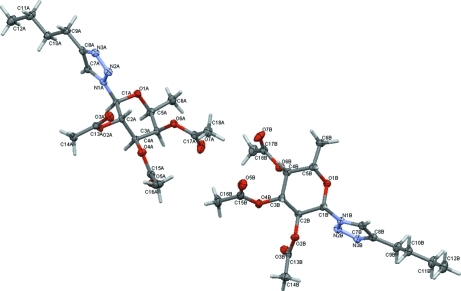
Thermal ellipsoid representation of both crystallographically independent molecules. Displacement ellipsoids are at the 50% level, hydrogen atoms are shown as spheres of arbitrary radii.

**Fig. 3.**
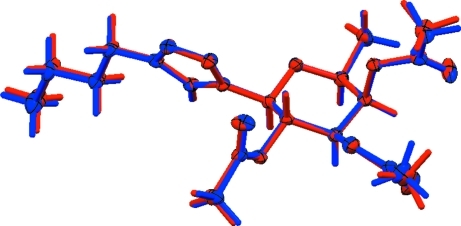
Overlay of the A and B molecules.

**Fig. 4.**
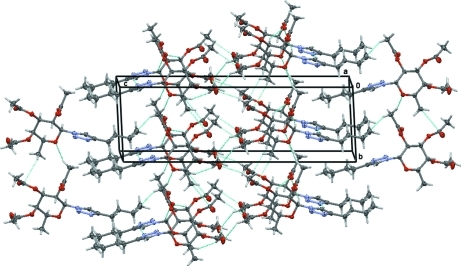
View of the packing arrangement. Blue dotted lines represent C—H···O and C—H···N interactions.

## supplementary crystallographic information

### Comment

*N*-Glycosidic analogs of naturally occurring carbohydrates are receiving a growing amount of attention due to their potential in medicinal chemistry (Norris, 2008; Temelkoff *et al.*, 2006). As part of a study into the synthesis of *N*-glycosyl-1,2,3-triazoles, the title compound was found to be the only 1,2,3-triazole product formed from the reaction of 2,3,4-tri-*O*-acetyl-β-L-fucopyranosyl azide (Zhang *et al.*, 2007) with 1-hexyne and catalytic CuSO_4_/ascorbic acid (Fig. 1).

The structure exhibits two crystallographically independent molecules A and B (Fig. 2) with essentially identical conformations as can be seen in the overlay shown in Fig. 3. The weighted r.m.s. deviation of the two molecules is only 0.09 Å. Both molecules exhibit unexceptional chair conformations for the pyranose ring and straight all-*trans* chains for the butyl chains. The crystal structure reveals the β-configuration of the pyranose *N*-glycoside (Fig. 2). This confirms the retention of stereochemistry during heterocycle formation with the *N*-glycosyl triazole group occupying the equatorial position at the anomeric carbon atom. Also, the complete regioselectivity of the cycloaddition process is supported with only the 1,4–1*H*-1,2,3-triazole being formed as the ^1^H NMR spectrum of the crude reaction mixture did not show any additional signals that may indicate the formation of the corresponding 1,5-isomer.

The molecules arrange in the solid state in layers with mainly hydrophobic sections built from the butyl triazole units on the one hand and the more polar moieties dominated by the carbohydrate units on the other. Within the polar layers intermolecular interactions are dominated by a three-dimensional network of weak C—H···O hydrogen bonds with the acetyl keto oxygen atoms as the H bond acceptors (Fig. 4, Table 1). The triazole units interact with each other *via* C—H···N hydrogen bonds that connect the molecules into two infinite chains made up of either A molecules or B molecules that stretch parallel to each other along the [1 0 0] direction (Figure 4, Table 1). In the hydrophobic layer dominated by the butyl groups no directional interactions are observed.

### Experimental

The triazole was prepared from 2,3,4-tri-*O*-acetyl-β-*L*-fucosyl azide (0.4 g, 1.27 mmol), 1-hexyne (0.16 ml, 1.38 mmol), 1*M* CuSO_4_ (0.3 ml, 0.3 mmol), 1*M* ascorbic acid (0.4 ml, 0.4 mmol) and 10 ml of 1:1 ethanol/H_2_O as solvent. The mixture was heated to 345.5 K (70 °C) and allowed to stir vigorously until TLC showed the completion of the reaction. The reaction was monitored by TLC (1:1, hexane-ethyl acetate, *R*_f_ = 0.41). After cooling to room temperature, ice water was added to the mixture which led to the precipitation of the triazole product which was then isolated by filtration through a glass frit. Purification by flash column chromatography (1:1, hexane-ethyl acetate) and recrystallization with isopropanol gave the title compound as a white solid (0.42 g, 83.3%). Crystals suitable for data collection were grown by slow evaporation from isopropanol. ^1^H NMR (CDCl_3_): δ 0.91 (t, 3H, *J* = 7.32 Hz), 1.23 (d, 3H, *J* = 6.22 Hz), 1.35 (m, 2H, *J* = 7.32 Hz), 1.64 (m, 2H, *J* = 7.32 Hz), 1.85 (s, 3H, COCH_3_), 1.98 (s, 3H, COCH_3_), 2.22 (s, 3H, COCH_3_), 2.70 (t, 2H, *J* = 7.32 Hz), 4.09 (q, 1H, *J* = 6.59 Hz), 5.21 (dd, 1H, *J* = 2.93, 10.25 Hz), 5.37 (d, 1H, *J* = 3.30 Hz), 5.50 (t, 1H, H-2, *J* = 9.89 Hz), 5.76 (d, 1H, H-1, *J* = 9.89 Hz), 7.54 (s, 1H, H-triazole); ^13^C NMR (CDCl_3_): δ 15.08, 17.33, 21.55, 21.83, 21.96, 23.45, 26.56, 32.53, 69.01, 71.07, 72.45, 73.75, 87.32, 119.86, 150.02, 170.20, 170.90, 171.39; MS: m/*z* calculated: 397.18, m/*z* found (ESI): 420.2 (+Na).

### Refinement

Treatment of hydrogen atoms: All hydrogen atoms were added in calculated positions with a C—H bond distance of 0.95 Å (triazole H), 0.98 Å (methyl) or 1.00 Å (others). They were refined with isotropic displacement parameters of 1.5 times (methyl) or 1.2 times (others) that of the equivalent isotropic displacement parameter of the adjacent carbon atom. Methyl hydrogen atoms were allowed to rotate to best fit the experimental electron density.

Friedel pairs were merged prior to refinement. The absolute structure was assigned based on the known stereochemistry of carbon atoms not being changed during the synthesis of the compound.

### Crystal data

**Table:**

C_18_H_27_N_3_O_7_	*Z* = 2
*M**_r_* = 397.43	*F*(000) = 424
Triclinic, *P*1	*D*_x_ = 1.294 Mg m^−^^3^
Hall symbol: P 1	Mo *K*α radiation, λ = 0.71073 Å
*a* = 5.5173 (3) Å	Cell parameters from 9354 reflections
*b* = 7.7442 (4) Å	θ = 2.6–30.5°
*c* = 24.1013 (13) Å	µ = 0.10 mm^−^^1^
α = 94.507 (1)°	*T* = 100 K
β = 96.151 (1)°	Plate, colourless
γ = 91.227 (1)°	0.36 × 0.35 × 0.09 mm
*V* = 1020.22 (9) Å^3^	

### Data collection

**Table:**

Bruker SMART APEX CCD diffractometer	5041 independent reflections
Radiation source: fine-focus sealed tube	4839 reflections with *I* > 2σ(*I*)
graphite	*R*_int_ = 0.020
ω scans	θ_max_ = 28.3°, θ_min_ = 0.9°
Absorption correction: multi-scan (*SADABS* in *SAINT-Plus*; Bruker, 2003)	*h* = −7→7
*T*_min_ = 0.867, *T*_max_ = 0.991	*k* = −10→10
10512 measured reflections	*l* = −31→32

### Refinement

**Table:**

Refinement on *F*^2^	Primary atom site location: structure-invariant direct methods
Least-squares matrix: full	Secondary atom site location: difference Fourier map
*R*[*F*^2^ > 2σ(*F*^2^)] = 0.046	Hydrogen site location: inferred from neighbouring sites
*wR*(*F*^2^) = 0.120	H-atom parameters constrained
*S* = 1.11	*w* = 1/[σ^2^(*F*_o_^2^) + (0.0771*P*)^2^ + 0.17*P*] where *P* = (*F*_o_^2^ + 2*F*_c_^2^)/3
5041 reflections	(Δ/σ)_max_ < 0.001
515 parameters	Δρ_max_ = 0.42 e Å^−^^3^
3 restraints	Δρ_min_ = −0.21 e Å^−^^3^

### Special details

**Table:**

Geometry. All e.s.d.'s (except the e.s.d. in the dihedral angle between two l.s. planes) are estimated using the full covariance matrix. The cell e.s.d.'s are taken into account individually in the estimation of e.s.d.'s in distances, angles and torsion angles; correlations between e.s.d.'s in cell parameters are only used when they are defined by crystal symmetry. An approximate (isotropic) treatment of cell e.s.d.'s is used for estimating e.s.d.'s involving l.s. planes.
Refinement. Refinement of *F*^2^ against ALL reflections. The weighted *R*-factor *wR* and goodness of fit *S* are based on *F*^2^, conventional *R*-factors *R* are based on *F*, with *F* set to zero for negative *F*^2^. The threshold expression of *F*^2^ > σ(*F*^2^) is used only for calculating *R*-factors(gt) *etc*. and is not relevant to the choice of reflections for refinement. *R*-factors based on *F*^2^ are statistically about twice as large as those based on *F*, and *R*- factors based on ALL data will be even larger.

### Fractional atomic coordinates and isotropic or equivalent isotropic displacement parameters (Å^2^)

**Table:**

	*x*	*y*	*z*	*U*_iso_*/*U*_eq_	
C1A	0.1284 (4)	0.5904 (3)	0.25737 (10)	0.0160 (4)	
H1A	0.2899	0.5447	0.2490	0.019*	
C2A	0.0093 (4)	0.4715 (3)	0.29447 (10)	0.0160 (4)	
H2A	−0.1530	0.5159	0.3027	0.019*	
C3A	0.1762 (4)	0.4617 (3)	0.34855 (10)	0.0172 (4)	
H3A	0.3249	0.3967	0.3405	0.021*	
C4A	0.2522 (4)	0.6418 (3)	0.37653 (10)	0.0178 (5)	
H4A	0.3898	0.6301	0.4064	0.021*	
C5A	0.3353 (5)	0.7604 (3)	0.33445 (11)	0.0201 (5)	
H5A	0.4950	0.7206	0.3229	0.024*	
C6A	0.3639 (6)	0.9487 (4)	0.35723 (13)	0.0282 (6)	
H6A1	0.4299	1.0173	0.3295	0.042*	
H6A2	0.4757	0.9583	0.3919	0.042*	
H6A3	0.2045	0.9923	0.3650	0.042*	
C7A	0.0505 (5)	0.6379 (3)	0.15536 (11)	0.0186 (5)	
H7A	0.2127	0.6396	0.1455	0.022*	
C8A	−0.1595 (4)	0.6638 (3)	0.12178 (11)	0.0189 (5)	
C9A	−0.1947 (5)	0.7003 (4)	0.06140 (12)	0.0238 (5)	
H9A1	−0.2156	0.8262	0.0590	0.029*	
H9A2	−0.3463	0.6399	0.0434	0.029*	
C10A	0.0155 (5)	0.6439 (4)	0.02915 (11)	0.0226 (5)	
H10A	0.0396	0.5186	0.0323	0.027*	
H10B	0.1665	0.7070	0.0463	0.027*	
C11A	−0.0269 (5)	0.6775 (5)	−0.03262 (12)	0.0307 (6)	
H11A	−0.1812	0.6180	−0.0494	0.037*	
H11B	−0.0451	0.8034	−0.0357	0.037*	
C12A	0.1782 (6)	0.6156 (5)	−0.06548 (13)	0.0351 (7)	
H12A	0.3314	0.6749	−0.0494	0.053*	
H12B	0.1425	0.6416	−0.1046	0.053*	
H12C	0.1936	0.4903	−0.0637	0.053*	
C13A	−0.2440 (4)	0.2243 (3)	0.26034 (11)	0.0192 (5)	
C14A	−0.2384 (5)	0.0438 (3)	0.23312 (12)	0.0248 (5)	
H14A	−0.1490	−0.0299	0.2590	0.037*	
H14B	−0.1569	0.0458	0.1990	0.037*	
H14C	−0.4056	−0.0026	0.2235	0.037*	
C15A	0.1526 (5)	0.3080 (3)	0.43018 (12)	0.0234 (5)	
C16A	−0.0247 (6)	0.2183 (5)	0.46177 (14)	0.0367 (7)	
H16A	0.0645	0.1544	0.4906	0.055*	
H16B	−0.1292	0.1373	0.4359	0.055*	
H16C	−0.1257	0.3043	0.4795	0.055*	
C17A	0.0802 (5)	0.7476 (3)	0.45847 (11)	0.0207 (5)	
C18A	−0.1564 (5)	0.7616 (4)	0.48340 (13)	0.0286 (6)	
H18A	−0.1241	0.7955	0.5236	0.043*	
H18B	−0.2447	0.6494	0.4777	0.043*	
H18C	−0.2553	0.8492	0.4653	0.043*	
N1A	−0.0240 (4)	0.6093 (3)	0.20568 (9)	0.0166 (4)	
N2A	−0.2688 (4)	0.6186 (3)	0.20427 (10)	0.0204 (4)	
N3A	−0.3509 (4)	0.6499 (3)	0.15312 (10)	0.0203 (4)	
O1A	0.1606 (3)	0.7576 (2)	0.28548 (8)	0.0195 (4)	
O2A	−0.0188 (3)	0.3011 (2)	0.26588 (8)	0.0181 (3)	
O3A	−0.4211 (3)	0.2926 (3)	0.27577 (9)	0.0262 (4)	
O4A	0.0358 (3)	0.3647 (2)	0.38316 (8)	0.0216 (4)	
O5A	0.3664 (4)	0.3301 (3)	0.44336 (9)	0.0329 (5)	
O6A	0.0444 (3)	0.7086 (2)	0.40218 (8)	0.0205 (4)	
O7A	0.2785 (4)	0.7661 (4)	0.48414 (9)	0.0385 (6)	
C1B	0.2703 (4)	0.9784 (3)	0.80834 (10)	0.0174 (5)	
H1B	0.1076	0.9344	0.8168	0.021*	
C2B	0.3807 (4)	0.8444 (3)	0.76952 (11)	0.0166 (4)	
H2B	0.5439	0.8862	0.7608	0.020*	
C3B	0.2060 (4)	0.8121 (3)	0.71631 (10)	0.0186 (5)	
H3B	0.0553	0.7500	0.7248	0.022*	
C4B	0.1364 (4)	0.9804 (3)	0.69039 (11)	0.0193 (5)	
H4B	−0.0028	0.9557	0.6605	0.023*	
C5B	0.0619 (5)	1.1164 (4)	0.73393 (11)	0.0212 (5)	
H5B	−0.0975	1.0789	0.7460	0.025*	
C6B	0.0375 (6)	1.2960 (4)	0.71358 (12)	0.0275 (6)	
H6B1	−0.0147	1.3756	0.7434	0.041*	
H6B2	−0.0838	1.2920	0.6806	0.041*	
H6B3	0.1953	1.3367	0.7036	0.041*	
C7B	0.3525 (5)	1.0728 (3)	0.91047 (11)	0.0195 (5)	
H7B	0.1904	1.0772	0.9203	0.023*	
C8B	0.5642 (4)	1.1177 (3)	0.94397 (11)	0.0187 (5)	
C9B	0.6021 (5)	1.1841 (4)	1.00422 (12)	0.0244 (5)	
H9B1	0.6318	1.3111	1.0067	0.029*	
H9B2	0.7496	1.1320	1.0222	0.029*	
C10B	0.3876 (5)	1.1448 (4)	1.03638 (11)	0.0232 (5)	
H10C	0.2409	1.1995	1.0190	0.028*	
H10D	0.3552	1.0180	1.0332	0.028*	
C11B	0.4303 (6)	1.2089 (5)	1.09785 (12)	0.0309 (6)	
H11C	0.4583	1.3361	1.1010	0.037*	
H11D	0.5800	1.1568	1.1149	0.037*	
C12B	0.2200 (6)	1.1658 (5)	1.13071 (13)	0.0351 (7)	
H12D	0.0720	1.2198	1.1149	0.053*	
H12E	0.2593	1.2100	1.1699	0.053*	
H12F	0.1931	1.0399	1.1285	0.053*	
C13B	0.6276 (5)	0.6187 (3)	0.80445 (11)	0.0192 (5)	
C14B	0.6181 (5)	0.4532 (4)	0.83252 (13)	0.0264 (6)	
H14D	0.5291	0.3635	0.8068	0.040*	
H14E	0.5345	0.4719	0.8663	0.040*	
H14F	0.7844	0.4160	0.8428	0.040*	
C15B	0.1945 (6)	0.6211 (4)	0.63345 (12)	0.0272 (6)	
C16B	0.3516 (7)	0.5229 (5)	0.59592 (14)	0.0402 (8)	
H16D	0.2618	0.4193	0.5779	0.060*	
H16E	0.4996	0.4886	0.6181	0.060*	
H16F	0.3965	0.5967	0.5672	0.060*	
C17B	0.3086 (5)	1.0620 (4)	0.60971 (11)	0.0218 (5)	
C18B	0.5441 (5)	1.0822 (5)	0.58499 (13)	0.0306 (6)	
H18D	0.5115	1.1205	0.5472	0.046*	
H18E	0.6251	0.9709	0.5832	0.046*	
H18F	0.6498	1.1686	0.6084	0.046*	
N1B	0.4259 (4)	1.0209 (3)	0.86032 (9)	0.0166 (4)	
N2B	0.6712 (4)	1.0341 (3)	0.86152 (10)	0.0202 (4)	
N3B	0.7542 (4)	1.0919 (3)	0.91256 (9)	0.0201 (4)	
O1B	0.2423 (3)	1.1344 (2)	0.78221 (8)	0.0195 (4)	
O2B	0.4016 (3)	0.6857 (2)	0.79666 (8)	0.0187 (3)	
O3B	0.8064 (3)	0.6837 (3)	0.78989 (10)	0.0285 (4)	
O4B	0.3322 (4)	0.7019 (2)	0.67849 (8)	0.0228 (4)	
O5B	−0.0221 (4)	0.6326 (3)	0.62559 (10)	0.0363 (5)	
O6B	0.3468 (3)	1.0408 (2)	0.66523 (8)	0.0209 (4)	
O7B	0.1098 (4)	1.0641 (4)	0.58456 (9)	0.0352 (5)	

### Atomic displacement parameters (Å^2^)

**Table:**

	*U*^11^	*U*^22^	*U*^33^	*U*^12^	*U*^13^	*U*^23^
C1A	0.0121 (10)	0.0187 (11)	0.0174 (11)	0.0013 (8)	0.0022 (8)	0.0014 (9)
C2A	0.0137 (10)	0.0157 (10)	0.0185 (11)	0.0005 (8)	0.0032 (8)	−0.0015 (8)
C3A	0.0154 (11)	0.0187 (11)	0.0183 (11)	0.0008 (8)	0.0032 (8)	0.0040 (9)
C4A	0.0139 (11)	0.0202 (11)	0.0191 (11)	0.0011 (9)	0.0026 (8)	−0.0017 (9)
C5A	0.0167 (11)	0.0218 (12)	0.0215 (12)	−0.0022 (9)	0.0013 (9)	0.0007 (9)
C6A	0.0322 (15)	0.0227 (13)	0.0281 (14)	−0.0071 (11)	−0.0006 (11)	−0.0002 (10)
C7A	0.0155 (11)	0.0213 (11)	0.0195 (12)	0.0015 (9)	0.0042 (9)	0.0003 (9)
C8A	0.0134 (11)	0.0216 (11)	0.0213 (12)	0.0008 (9)	0.0035 (9)	−0.0022 (9)
C9A	0.0177 (12)	0.0311 (14)	0.0230 (13)	0.0057 (10)	0.0015 (9)	0.0051 (10)
C10A	0.0212 (12)	0.0263 (13)	0.0209 (12)	0.0020 (10)	0.0039 (9)	0.0031 (10)
C11A	0.0249 (14)	0.0474 (18)	0.0208 (13)	0.0034 (12)	0.0035 (10)	0.0067 (12)
C12A	0.0313 (16)	0.0507 (19)	0.0253 (15)	0.0038 (13)	0.0087 (12)	0.0065 (13)
C13A	0.0171 (11)	0.0213 (12)	0.0192 (12)	0.0014 (9)	0.0006 (9)	0.0023 (9)
C14A	0.0240 (13)	0.0202 (12)	0.0288 (14)	0.0010 (10)	0.0005 (10)	−0.0029 (10)
C15A	0.0291 (14)	0.0182 (11)	0.0231 (13)	0.0025 (10)	0.0035 (10)	0.0021 (9)
C16A	0.0382 (18)	0.0403 (17)	0.0343 (16)	−0.0012 (14)	0.0052 (13)	0.0192 (13)
C17A	0.0157 (11)	0.0221 (12)	0.0243 (12)	0.0011 (9)	0.0034 (9)	−0.0006 (9)
C18A	0.0159 (12)	0.0402 (16)	0.0297 (14)	0.0012 (11)	0.0062 (10)	−0.0018 (12)
N1A	0.0123 (9)	0.0177 (9)	0.0199 (10)	0.0007 (7)	0.0017 (7)	0.0010 (7)
N2A	0.0121 (9)	0.0221 (10)	0.0270 (11)	−0.0006 (8)	0.0027 (8)	0.0018 (8)
N3A	0.0148 (10)	0.0221 (10)	0.0243 (11)	−0.0001 (8)	0.0037 (8)	0.0019 (8)
O1A	0.0183 (8)	0.0172 (8)	0.0222 (9)	−0.0004 (6)	−0.0001 (7)	0.0001 (7)
O2A	0.0133 (8)	0.0174 (8)	0.0233 (9)	0.0017 (6)	0.0033 (6)	−0.0017 (7)
O3A	0.0172 (9)	0.0231 (9)	0.0385 (11)	−0.0029 (7)	0.0079 (8)	−0.0019 (8)
O4A	0.0183 (8)	0.0242 (9)	0.0230 (9)	−0.0012 (7)	0.0035 (7)	0.0049 (7)
O5A	0.0300 (11)	0.0354 (12)	0.0319 (11)	0.0004 (9)	−0.0069 (9)	0.0089 (9)
O6A	0.0144 (8)	0.0229 (9)	0.0238 (9)	0.0038 (7)	0.0023 (7)	−0.0020 (7)
O7A	0.0173 (10)	0.0686 (17)	0.0267 (11)	0.0044 (10)	0.0018 (8)	−0.0144 (11)
C1B	0.0139 (10)	0.0202 (11)	0.0173 (11)	−0.0023 (9)	−0.0002 (8)	0.0009 (9)
C2B	0.0140 (10)	0.0161 (10)	0.0195 (11)	−0.0026 (8)	0.0013 (8)	0.0023 (8)
C3B	0.0180 (11)	0.0201 (11)	0.0171 (11)	−0.0015 (9)	0.0013 (8)	0.0005 (9)
C4B	0.0134 (11)	0.0241 (12)	0.0202 (12)	−0.0022 (9)	0.0008 (9)	0.0037 (9)
C5B	0.0164 (11)	0.0252 (12)	0.0219 (12)	0.0006 (9)	0.0003 (9)	0.0043 (9)
C6B	0.0311 (14)	0.0244 (13)	0.0266 (14)	0.0054 (11)	−0.0020 (11)	0.0046 (11)
C7B	0.0155 (11)	0.0209 (11)	0.0220 (12)	−0.0009 (9)	0.0032 (9)	0.0007 (9)
C8B	0.0144 (11)	0.0185 (11)	0.0232 (12)	−0.0022 (9)	0.0017 (9)	0.0033 (9)
C9B	0.0185 (12)	0.0321 (14)	0.0216 (13)	−0.0063 (10)	0.0013 (9)	−0.0007 (10)
C10B	0.0199 (12)	0.0279 (13)	0.0213 (12)	−0.0035 (10)	0.0029 (9)	−0.0011 (10)
C11B	0.0248 (14)	0.0444 (17)	0.0222 (14)	−0.0059 (12)	0.0040 (10)	−0.0046 (12)
C12B	0.0304 (16)	0.0494 (19)	0.0253 (14)	−0.0049 (14)	0.0091 (12)	−0.0043 (13)
C13B	0.0184 (12)	0.0193 (11)	0.0190 (12)	−0.0015 (9)	0.0016 (9)	−0.0029 (9)
C14B	0.0224 (13)	0.0248 (13)	0.0324 (15)	0.0004 (10)	0.0006 (10)	0.0071 (11)
C15B	0.0409 (17)	0.0186 (12)	0.0204 (13)	−0.0067 (11)	−0.0024 (11)	0.0012 (10)
C16B	0.058 (2)	0.0306 (15)	0.0298 (16)	−0.0025 (15)	0.0037 (14)	−0.0102 (12)
C17B	0.0167 (12)	0.0260 (13)	0.0228 (12)	0.0006 (9)	0.0043 (9)	0.0000 (10)
C18B	0.0187 (13)	0.0454 (17)	0.0281 (14)	−0.0006 (11)	0.0073 (11)	−0.0006 (12)
N1B	0.0121 (9)	0.0196 (10)	0.0179 (10)	−0.0014 (7)	0.0018 (7)	0.0005 (8)
N2B	0.0117 (9)	0.0252 (11)	0.0233 (11)	−0.0001 (8)	0.0014 (7)	0.0003 (8)
N3B	0.0146 (9)	0.0244 (11)	0.0212 (10)	0.0009 (8)	0.0023 (8)	0.0008 (8)
O1B	0.0187 (8)	0.0184 (8)	0.0207 (9)	−0.0015 (6)	−0.0008 (7)	0.0015 (7)
O2B	0.0153 (8)	0.0194 (8)	0.0217 (9)	−0.0017 (6)	0.0017 (6)	0.0049 (7)
O3B	0.0175 (9)	0.0261 (10)	0.0432 (12)	0.0005 (7)	0.0081 (8)	0.0059 (9)
O4B	0.0266 (10)	0.0210 (9)	0.0200 (9)	−0.0018 (7)	0.0025 (7)	−0.0028 (7)
O5B	0.0365 (13)	0.0351 (12)	0.0329 (12)	−0.0066 (9)	−0.0092 (9)	−0.0049 (9)
O6B	0.0149 (8)	0.0252 (9)	0.0225 (9)	−0.0050 (7)	0.0006 (7)	0.0054 (7)
O7B	0.0190 (10)	0.0659 (16)	0.0212 (10)	−0.0011 (10)	0.0010 (7)	0.0101 (10)

### Geometric parameters (Å, °)

**Table:**

C1A—O1A	1.412 (3)	C1B—O1B	1.409 (3)
C1A—N1A	1.446 (3)	C1B—N1B	1.452 (3)
C1A—C2A	1.519 (3)	C1B—C2B	1.523 (3)
C1A—H1A	1.0000	C1B—H1B	1.0000
C2A—O2A	1.437 (3)	C2B—O2B	1.438 (3)
C2A—C3A	1.521 (3)	C2B—C3B	1.520 (3)
C2A—H2A	1.0000	C2B—H2B	1.0000
C3A—O4A	1.440 (3)	C3B—O4B	1.443 (3)
C3A—C4A	1.530 (3)	C3B—C4B	1.528 (3)
C3A—H3A	1.0000	C3B—H3B	1.0000
C4A—O6A	1.446 (3)	C4B—O6B	1.451 (3)
C4A—C5A	1.520 (4)	C4B—C5B	1.522 (4)
C4A—H4A	1.0000	C4B—H4B	1.0000
C5A—O1A	1.440 (3)	C5B—O1B	1.444 (3)
C5A—C6A	1.516 (4)	C5B—C6B	1.515 (4)
C5A—H5A	1.0000	C5B—H5B	1.0000
C6A—H6A1	0.9800	C6B—H6B1	0.9800
C6A—H6A2	0.9800	C6B—H6B2	0.9800
C6A—H6A3	0.9800	C6B—H6B3	0.9800
C7A—N1A	1.354 (3)	C7B—N1B	1.350 (3)
C7A—C8A	1.369 (3)	C7B—C8B	1.369 (3)
C7A—H7A	0.9500	C7B—H7B	0.9500
C8A—N3A	1.369 (3)	C8B—N3B	1.367 (3)
C8A—C9A	1.497 (4)	C8B—C9B	1.493 (4)
C9A—C10A	1.517 (4)	C9B—C10B	1.520 (4)
C9A—H9A1	0.9900	C9B—H9B1	0.9900
C9A—H9A2	0.9900	C9B—H9B2	0.9900
C10A—C11A	1.526 (4)	C10B—C11B	1.517 (4)
C10A—H10A	0.9900	C10B—H10C	0.9900
C10A—H10B	0.9900	C10B—H10D	0.9900
C11A—C12A	1.514 (4)	C11B—C12B	1.519 (4)
C11A—H11A	0.9900	C11B—H11C	0.9900
C11A—H11B	0.9900	C11B—H11D	0.9900
C12A—H12A	0.9800	C12B—H12D	0.9800
C12A—H12B	0.9800	C12B—H12E	0.9800
C12A—H12C	0.9800	C12B—H12F	0.9800
C13A—O3A	1.200 (3)	C13B—O3B	1.196 (3)
C13A—O2A	1.355 (3)	C13B—O2B	1.361 (3)
C13A—C14A	1.500 (4)	C13B—C14B	1.498 (4)
C14A—H14A	0.9800	C14B—H14D	0.9800
C14A—H14B	0.9800	C14B—H14E	0.9800
C14A—H14C	0.9800	C14B—H14F	0.9800
C15A—O5A	1.193 (4)	C15B—O5B	1.196 (4)
C15A—O4A	1.351 (3)	C15B—O4B	1.358 (3)
C15A—C16A	1.495 (4)	C15B—C16B	1.495 (5)
C16A—H16A	0.9800	C16B—H16D	0.9800
C16A—H16B	0.9800	C16B—H16E	0.9800
C16A—H16C	0.9800	C16B—H16F	0.9800
C17A—O7A	1.198 (3)	C17B—O7B	1.196 (3)
C17A—O6A	1.359 (3)	C17B—O6B	1.355 (3)
C17A—C18A	1.497 (4)	C17B—C18B	1.496 (4)
C18A—H18A	0.9800	C18B—H18D	0.9800
C18A—H18B	0.9800	C18B—H18E	0.9800
C18A—H18C	0.9800	C18B—H18F	0.9800
N1A—N2A	1.351 (3)	N1B—N2B	1.352 (3)
N2A—N3A	1.310 (3)	N2B—N3B	1.307 (3)
			
O1A—C1A—N1A	106.41 (19)	O1B—C1B—N1B	106.14 (19)
O1A—C1A—C2A	108.84 (19)	O1B—C1B—C2B	109.43 (19)
N1A—C1A—C2A	111.89 (19)	N1B—C1B—C2B	112.5 (2)
O1A—C1A—H1A	109.9	O1B—C1B—H1B	109.6
N1A—C1A—H1A	109.9	N1B—C1B—H1B	109.6
C2A—C1A—H1A	109.9	C2B—C1B—H1B	109.6
O2A—C2A—C1A	108.36 (19)	O2B—C2B—C3B	108.53 (19)
O2A—C2A—C3A	108.51 (19)	O2B—C2B—C1B	108.20 (19)
C1A—C2A—C3A	108.96 (19)	C3B—C2B—C1B	108.09 (19)
O2A—C2A—H2A	110.3	O2B—C2B—H2B	110.6
C1A—C2A—H2A	110.3	C3B—C2B—H2B	110.6
C3A—C2A—H2A	110.3	C1B—C2B—H2B	110.6
O4A—C3A—C2A	104.62 (19)	O4B—C3B—C2B	105.76 (19)
O4A—C3A—C4A	111.7 (2)	O4B—C3B—C4B	110.6 (2)
C2A—C3A—C4A	111.91 (19)	C2B—C3B—C4B	112.0 (2)
O4A—C3A—H3A	109.5	O4B—C3B—H3B	109.5
C2A—C3A—H3A	109.5	C2B—C3B—H3B	109.5
C4A—C3A—H3A	109.5	C4B—C3B—H3B	109.5
O6A—C4A—C5A	111.8 (2)	O6B—C4B—C5B	110.9 (2)
O6A—C4A—C3A	107.00 (19)	O6B—C4B—C3B	107.1 (2)
C5A—C4A—C3A	111.2 (2)	C5B—C4B—C3B	111.6 (2)
O6A—C4A—H4A	108.9	O6B—C4B—H4B	109.1
C5A—C4A—H4A	108.9	C5B—C4B—H4B	109.1
C3A—C4A—H4A	108.9	C3B—C4B—H4B	109.1
O1A—C5A—C6A	105.6 (2)	O1B—C5B—C6B	105.7 (2)
O1A—C5A—C4A	111.2 (2)	O1B—C5B—C4B	110.5 (2)
C6A—C5A—C4A	113.1 (2)	C6B—C5B—C4B	114.1 (2)
O1A—C5A—H5A	108.9	O1B—C5B—H5B	108.8
C6A—C5A—H5A	108.9	C6B—C5B—H5B	108.8
C4A—C5A—H5A	108.9	C4B—C5B—H5B	108.8
C5A—C6A—H6A1	109.5	C5B—C6B—H6B1	109.5
C5A—C6A—H6A2	109.5	C5B—C6B—H6B2	109.5
H6A1—C6A—H6A2	109.5	H6B1—C6B—H6B2	109.5
C5A—C6A—H6A3	109.5	C5B—C6B—H6B3	109.5
H6A1—C6A—H6A3	109.5	H6B1—C6B—H6B3	109.5
H6A2—C6A—H6A3	109.5	H6B2—C6B—H6B3	109.5
N1A—C7A—C8A	104.7 (2)	N1B—C7B—C8B	104.4 (2)
N1A—C7A—H7A	127.6	N1B—C7B—H7B	127.8
C8A—C7A—H7A	127.6	C8B—C7B—H7B	127.8
C7A—C8A—N3A	107.9 (2)	N3B—C8B—C7B	108.0 (2)
C7A—C8A—C9A	129.8 (2)	N3B—C8B—C9B	122.2 (2)
N3A—C8A—C9A	122.3 (2)	C7B—C8B—C9B	129.8 (2)
C8A—C9A—C10A	113.5 (2)	C8B—C9B—C10B	113.5 (2)
C8A—C9A—H9A1	108.9	C8B—C9B—H9B1	108.9
C10A—C9A—H9A1	108.9	C10B—C9B—H9B1	108.9
C8A—C9A—H9A2	108.9	C8B—C9B—H9B2	108.9
C10A—C9A—H9A2	108.9	C10B—C9B—H9B2	108.9
H9A1—C9A—H9A2	107.7	H9B1—C9B—H9B2	107.7
C9A—C10A—C11A	112.7 (2)	C11B—C10B—C9B	113.1 (2)
C9A—C10A—H10A	109.0	C11B—C10B—H10C	108.9
C11A—C10A—H10A	109.0	C9B—C10B—H10C	108.9
C9A—C10A—H10B	109.0	C11B—C10B—H10D	108.9
C11A—C10A—H10B	109.0	C9B—C10B—H10D	108.9
H10A—C10A—H10B	107.8	H10C—C10B—H10D	107.8
C12A—C11A—C10A	113.0 (2)	C10B—C11B—C12B	113.6 (3)
C12A—C11A—H11A	109.0	C10B—C11B—H11C	108.9
C10A—C11A—H11A	109.0	C12B—C11B—H11C	108.9
C12A—C11A—H11B	109.0	C10B—C11B—H11D	108.9
C10A—C11A—H11B	109.0	C12B—C11B—H11D	108.9
H11A—C11A—H11B	107.8	H11C—C11B—H11D	107.7
C11A—C12A—H12A	109.5	C11B—C12B—H12D	109.5
C11A—C12A—H12B	109.5	C11B—C12B—H12E	109.5
H12A—C12A—H12B	109.5	H12D—C12B—H12E	109.5
C11A—C12A—H12C	109.5	C11B—C12B—H12F	109.5
H12A—C12A—H12C	109.5	H12D—C12B—H12F	109.5
H12B—C12A—H12C	109.5	H12E—C12B—H12F	109.5
O3A—C13A—O2A	124.0 (2)	O3B—C13B—O2B	123.8 (2)
O3A—C13A—C14A	125.5 (2)	O3B—C13B—C14B	125.6 (2)
O2A—C13A—C14A	110.5 (2)	O2B—C13B—C14B	110.6 (2)
C13A—C14A—H14A	109.5	C13B—C14B—H14D	109.5
C13A—C14A—H14B	109.5	C13B—C14B—H14E	109.5
H14A—C14A—H14B	109.5	H14D—C14B—H14E	109.5
C13A—C14A—H14C	109.5	C13B—C14B—H14F	109.5
H14A—C14A—H14C	109.5	H14D—C14B—H14F	109.5
H14B—C14A—H14C	109.5	H14E—C14B—H14F	109.5
O5A—C15A—O4A	123.3 (3)	O5B—C15B—O4B	123.1 (3)
O5A—C15A—C16A	126.7 (3)	O5B—C15B—C16B	126.2 (3)
O4A—C15A—C16A	110.0 (2)	O4B—C15B—C16B	110.7 (3)
C15A—C16A—H16A	109.5	C15B—C16B—H16D	109.5
C15A—C16A—H16B	109.5	C15B—C16B—H16E	109.5
H16A—C16A—H16B	109.5	H16D—C16B—H16E	109.5
C15A—C16A—H16C	109.5	C15B—C16B—H16F	109.5
H16A—C16A—H16C	109.5	H16D—C16B—H16F	109.5
H16B—C16A—H16C	109.5	H16E—C16B—H16F	109.5
O7A—C17A—O6A	123.1 (2)	O7B—C17B—O6B	123.2 (2)
O7A—C17A—C18A	125.3 (3)	O7B—C17B—C18B	125.3 (3)
O6A—C17A—C18A	111.6 (2)	O6B—C17B—C18B	111.5 (2)
C17A—C18A—H18A	109.5	C17B—C18B—H18D	109.5
C17A—C18A—H18B	109.5	C17B—C18B—H18E	109.5
H18A—C18A—H18B	109.5	H18D—C18B—H18E	109.5
C17A—C18A—H18C	109.5	C17B—C18B—H18F	109.5
H18A—C18A—H18C	109.5	H18D—C18B—H18F	109.5
H18B—C18A—H18C	109.5	H18E—C18B—H18F	109.5
N2A—N1A—C7A	111.1 (2)	C7B—N1B—N2B	111.4 (2)
N2A—N1A—C1A	121.4 (2)	C7B—N1B—C1B	126.5 (2)
C7A—N1A—C1A	127.1 (2)	N2B—N1B—C1B	121.5 (2)
N3A—N2A—N1A	106.6 (2)	N3B—N2B—N1B	106.3 (2)
N2A—N3A—C8A	109.6 (2)	N2B—N3B—C8B	109.8 (2)
C1A—O1A—C5A	111.88 (18)	C1B—O1B—C5B	111.96 (19)
C13A—O2A—C2A	117.27 (19)	C13B—O2B—C2B	117.18 (19)
C15A—O4A—C3A	117.9 (2)	C15B—O4B—C3B	116.7 (2)
C17A—O6A—C4A	115.87 (19)	C17B—O6B—C4B	115.64 (19)

### Hydrogen-bond geometry (Å, °)

**Table:**

*D*—H···*A*	*D*—H	H···*A*	*D*···*A*	*D*—H···*A*
C1B—H1B···O3B^i^	1.00	2.53	3.362 (3)	141
C2A—H2A···O3A	1.00	2.26	2.701 (3)	105
C2B—H2B···O3B	1.00	2.26	2.698 (3)	105
C3A—H3A···O3A^ii^	1.00	2.32	3.214 (3)	149
C3B—H3B···O3B^i^	1.00	2.27	3.165 (3)	148
C4A—H4A···O5A	1.00	2.56	3.046 (3)	110
C4A—H4A···O7A	1.00	2.23	2.682 (3)	106
C4B—H4B···O5B	1.00	2.57	3.067 (3)	110
C4B—H4B···O7B	1.00	2.21	2.670 (3)	106
C7A—H7A···N3A^ii^	0.95	2.39	3.308 (4)	161
C7B—H7B···N3B^i^	0.95	2.40	3.313 (4)	161
C14A—H14A···O1A^iii^	0.98	2.47	3.377 (3)	154
C14B—H14D···O1B^iii^	0.98	2.35	3.261 (3)	155
C16A—H16A···O7B^iii^	0.98	2.41	3.295 (4)	150
C16B—H16F···O7A	0.98	2.51	3.401 (4)	150

Symmetry codes: (i) *x*−1, *y*, *z*; (ii) *x*+1, *y*, *z*; (iii) *x*, *y*−1, *z*.
